# Combined effects of green tea supplementation and eccentric exercise on nuclear factor erythroid 2-related factor 2 activity

**DOI:** 10.1007/s00421-023-05271-8

**Published:** 2023-07-13

**Authors:** Josh Thorley, Craig Thomas, Nicolas Thon, Hannah Nuttall, Neil R. W. Martin, Nicolette Bishop, Stephen J. Bailey, Tom Clifford

**Affiliations:** School of Sport, Exercise and Health Sciences, Loughborough University, Loughborough, LE11 3TU UK

**Keywords:** Polyphenols, Antioxidants, Oxidative stress, Redox balance, Exercise

## Abstract

**Purpose:**

This study investigated whether combining eccentric exercise and green tea supplementation synergistically increased nuclear factor erythroid 2-related factor 2 (NRF2) activity, a transcription factor responsible for coordinating endogenous antioxidant expression.

**Methods:**

In a double-blinded, randomized, between-subjects design, 24 males (mean [SD]; 23 [3] years, 179.6 [6.1] cm, 78.8 [10.6] kg) performed 100 drop jumps following a 6 days supplementation period with either green tea (poly)phenols (*n* = 12; 500 mg·d^−1^) or a placebo (*n* = 12; inulin). NRF2/antioxidant response element (ARE) binding in peripheral blood mononuclear cells (PBMCs), catalase (CAT) and glutathione reductase (GR) activity, 8-hydroxy-2′-deoxyguanosine (8-OHdG) excretion, and differential leukocyte counts were measured pre-, post-, 1 h and 24 h post-exercise.

**Results:**

Exercise did not increase NRF2/ARE binding (*p* = 0.12) (fold change vs rest: green tea = [post] 0.78 ± 0.45, [1 h] 1.17 ± 0.54, [24 h] 1.06 ± 0.56; placebo = [post] 1.40 ± 1.50, [1 h] 2.98 ± 3.70, [24 h] 1.04 ± 0.45). Furthermore, CAT activity (*p* = 0.12) and 8-OHdG excretion (*p* = 0.42) were unchanged in response to exercise and were not augmented by green tea supplementation (*p* > 0.05 for all). Exercise increased GR activity by 30% (*p* = 0.01), however no differences were found between supplement groups (*p* = 0.51). Leukocyte and neutrophil concentrations were only elevated post-exercise (*p* < 0.001 for all).

**Conclusion:**

Eccentric exercise, either performed alone or in conjunction with green tea supplementation, did not significantly increase NRF2 activity in PBMCs.

**Trial registration number:**

osf.io/kz37g (registered: 15/09/21).

**Graphical abstract:**

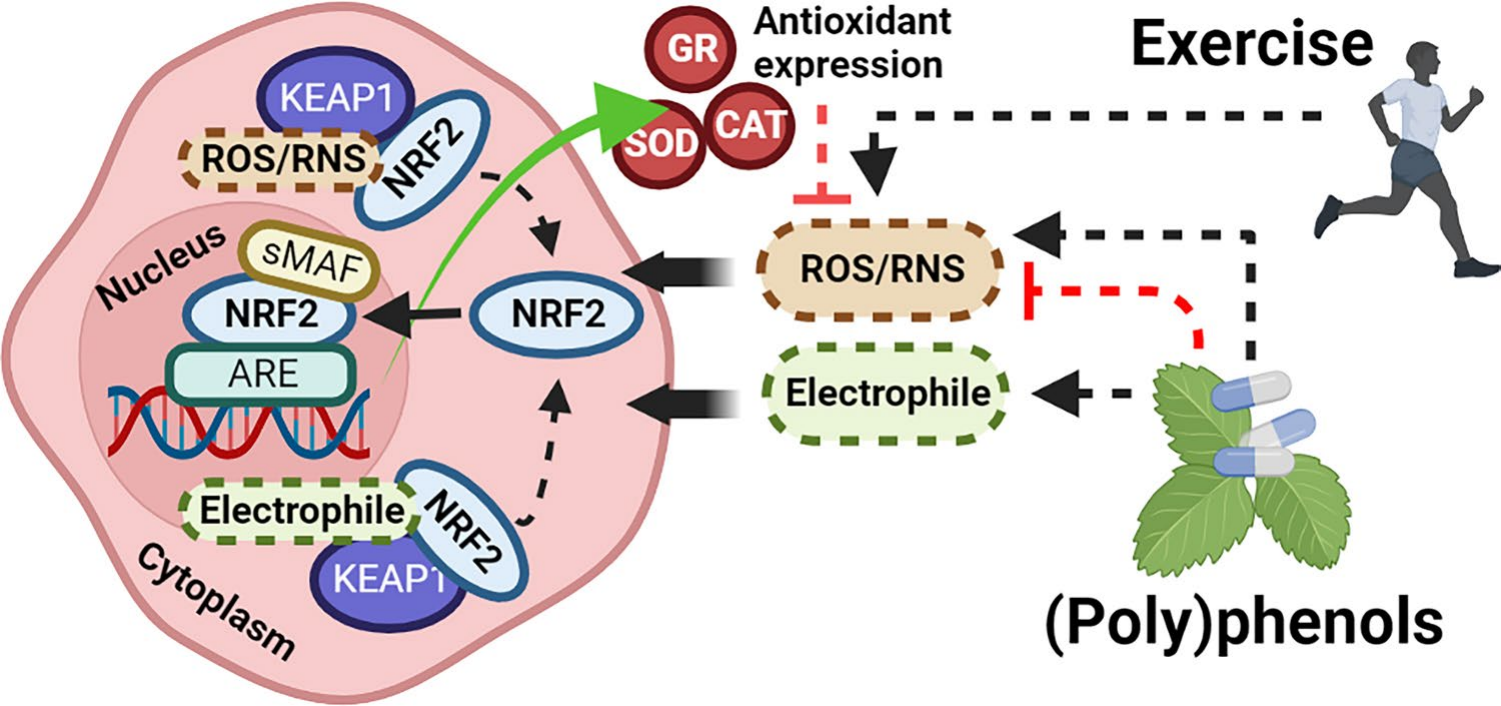

## Introduction

Endogenous enzymatic antioxidants including catalase (CAT) and glutathione reductase (GR) maintain redox balance and suppress oxidative damage by scavenging reactive oxygen and nitrogen species (RONS) such as hydrogen peroxide (H_2_O_2_), superoxide (O_2_^•–^), peryoxynitrite, and hydroxyl radical (Sies 2017). These antioxidant enzymes are primarily synthesized through the activation of nuclear factor erythroid 2-related factor 2 (NRF2), a thiol-sensitive transcription factor triggered by oxidative and/or electrophilic challenge (Dinkova-Kostova et al. 2005). At rest in healthy individuals, NRF2 is continually ubiquitinated and degraded by the cysteine rich inhibitor protein kelch-like ECH-associated protein 1 (KEAP1) in the cytosol (Dinkova-Kostova et al. 2017). Canonical activation of NRF2 occurs when oxidative or electrophilic stress covalently modifies KEAP1 cysteine thiol residues, inhibiting NRF2 ubiquitylation and enabling NRF2 accumulation in the nucleus. Following heterodimerization with small musculoaponeurotic fibrosarcoma proteins, NRF2 binds to antioxidant response elements (ARE) on the promotor region of target genes, increasing the expression of antioxidant enzymes (Wasserman and Fahl 1997; Hirotsu et al. 2012). NRF2 can also be activated via non-canonical pathways, where signaling proteins including extracellular signal-regulated kinase 1/2 (ERK1/2) can disrupt the KEAP1-NRF2 complex (Verma et al. 2015; Yang et al. 2019).

There is a growing interest in strategies to activate NRF2 given the important role antioxidant enzymes play in mitigating oxidative distress and inflammation (Liguori et al. 2018; Cuadrado et al. 2019). Recent research suggests exercise may activate NRF2, presumably by increasing RONS production from contracting skeletal muscle (Done and Traustadóttir 2016). In skeletal muscle, NRF2 gene expression was increased 2.5 h following 30 min of moderate-intensity treadmill running in middle-aged, recreationally trained, females (Scott et al. 2015), and 6 h following 90 min of high intensity interval cycling in young males (Ballmann et al. 2014). In peripheral blood mononuclear cells (PBMCs), Done et al. (2016) reported that 10 min following 30 min cycling at 70% $\dot{V}{\text{O}}_{{{\text{2max}}}}$, NRF2 whole-cell protein content increased in young and older males, with nuclear import of NRF2 being impaired in the older group. They later expanded these findings, reporting that steady state (30 min cycling at 70% $\dot{V}{\text{O}}_{{{\text{2max}}}}$) and high-intensity interval cycling (7 high intensity intervals, each 1 min duration at 90% $\dot{V}{\text{O}}_{{{\text{2max}}}}$) increased NRF2 protein content, but there were no differences between exercise modalities (Done et al. 2017). As demonstrated, much of the work reporting NRF2 activation post-exercise in humans has used aerobic, concentric exercise (i.e., cycling). Lower metabolically demanding types of exercise, such as eccentric exercise, is also frequently used to induce a variety of exercise adaptations in a range of populations (Harris-Love et al. 2021), however the potential of eccentric exercise to augment NRF2 has not yet been fully explored.

Another strategy that could increase NRF2 activity is (poly)phenol supplementation. (Poly)phenols, chemical compounds abundant in plants, have been shown to activate NRF2, ostensibly via electrophilic and/or oxidative modification of KEAP1 (Satoh et al. 2013; Eghbaliferiz and Iranshahi 2016). Indeed, in vitro and in vivo rodent studies have reported that (poly)phenol compounds in red wine (resveratrol), olive oil (hydroxytyrosol), and coffee (caffeic acid) activate NRF2 and increase the expression of antioxidant enzymes (Narayanan et al. 2015; Bigagli et al. 2017; Shen et al. 2018). Furthermore, a wealth of research has indicated that epigallocatechin gallate (EGCG), a catechin-type (poly)phenol found abundantly in certain teas, fruits, and nuts, can activate NRF2 in vitro and in vivo using rodents (Zheng et al. 2012; Han et al. 2012; Ye et al. 2015; Li et al. 2016; Sun et al. 2017; Tian et al. 2021). However, few studies have examined the effects of (poly)phenols on NRF2 activity in humans, with none examining the effect of sources high in EGCG (Clifford et al. 2021).

Despite some evidence supporting exercise and (poly)phenols as independent NRF2 activators, their synergistic effects have received scant attention. Combining these two interventions may further augment NRF2 activation, since the oxidative and electrophilic compounds generated by exercise and (poly)phenols may react with entirely separate KEAP1-specific cysteine (Cys) residues to suppress NRF2 ubiquitylation (i.e., Cys^151^, Cys^273^, Cys^288^ by electrophiles; Cys^226^, Cys^613^, Cys^622/624^ by H_2_O_2_) (Suzuki et al. 2019). In addition, eccentric exercise may not generate a sufficient pro-oxidative stimulus to enable canonical NRF2 activation (Kamandulis et al. 2017) and could subsequently benefit from other activators, such as (poly)phenols, to maximize this response.

There is some evidence of synergistic activation in rats; for example, Sahin et al. (2016) found that combining curcumin supplementation with treadmill running (5 d.wk for 6 weeks) increased NRF2 protein content to a greater extent than exercise or curcumin alone. Only one study has explored the potential synergistic effect of eccentric exercise (10 × 30 maximal eccentric knee flexion contraction) and (poly)phenols in humans, and although this was only a secondary outcome, they found no differences in post- or 24 h post-exercise skeletal muscle NRF2 protein content following a 7 days supplementation period with a (poly)phenol rich tart cherry juice (Wangdi et al. 2021). Given the important role of NRF2 in health and disease, the potential synergistic effects of (poly)phenols and exercise on NRF2 activity warrants further investigation.

The primary aim of this study was to determine whether (poly)phenol supplementation for 6 days before eccentric exercise would lead to greater NRF2 activity in PBMCs than exercise alone. This was the first study to measure NRF2 by quantifying the binding of nuclear-bound NRF2 from PBMCs to ARE oligonucleotides, a novel method yet to be employed in a randomized control trial with exercise. A secondary aim was to measure the downstream activity of NRF2 targets CAT and GR, and levels of oxidative damage via measurement of 8-hydroxy-2′-deoxyguanosine (8-OHdG). This study used green tea as the (poly)phenol intervention due to its superior bioavailability and high concentration of EGCG (Singh et al. 2011). We hypothesized that combining eccentric exercise with 6 days of green tea supplementation would augment NRF2 activity to a greater extent than eccentric exercise alone, and that this would have favorable downstream effects on NRF2-target antioxidant activity and oxidative damage.

## Methods

### Participants

Twenty-four male participants (mean [SD]; 23 [3] years, 179.6 [6.1] cm, 78.8 [10.6] kg) who were classified as recreationally active according to a recently published participant classification framework (McKay et al. 2021) were recruited for this study. Sample size was determined by a simulation-based power analysis for our primary outcome measure, changes in NRF2/ARE binding, using the ANOVA_Power shiny app (Lakens and Caldwell 2021). No other study has performed this analysis in humans; thus, power analysis was performed using mean and SD data from a study using similar analytical methods in rodents (Ostrom and Traustadóttir 2020). This analysis indicated that, with a difference in means and SD of 0.01 and 0.0075 units, respectively, 11 participants per group would provide ≥ 80% power to detect a time and interaction effect (effect size of ≥ 0.22 [partial eta squared]). As such, we successfully recruited 22 participants to undertake this investigation. However, due to technical difficulties with the NRF2/ARE binding assay, we lost data from 2 participants (n = 1 per group) and thus to regain statistical power, we recruited 1 more participant for each group.

In line with the definition of ‘recreationally active’, participants were undertaking at least ≥ 3 sessions of moderate-intensity physical activity (for ≥ 30 min each session), including ≥ 2 resistance training sessions, per week. Participants completed a health screening survey to determine their eligibility; any with a history of or current cardiovascular or metabolic disease, had a musculoskeletal injury, had a food allergy, or were taking medication, were excluded from participating. Trained individuals, defined as those completing > 4 resistance training sessions per week in the previous 6 months were excluded from the study as their familiarity with similar exercise stressors may have attenuated the disruption to redox status. For the duration of the study, participants were instructed to refrain from using any putative recovery interventions such as ice baths or consuming any dietary supplements. Muscle-damaging exercise (i.e., high volume and/or high intensity resistance or aerobic exercise) was restricted in the 48 h prior to the second visit until completion of the study.

### Experimental design

This study employed a double-blinded, placebo-controlled, between-subjects design and data was collected at the National Centre for Sport and Exercise Medicine (East Midlands), Loughborough University between April 2021 and January 2023. Ethical approval was granted by Loughborough University Research Ethics Committee, Human Participants Sub-committee, and the study was pre-registered on the Open Science Framework prior to data collection (osf.io/kz37g). Participants provided written informed consent and all procedures conformed to the guidance presented by the Declaration of Helsinki.

Participants were randomized to a placebo (*n* = 12) or a green tea (*n* = 12) supplement group using minimization randomization; this was based on their maximal counter movement jump (CMJ) height recorded at familiarization. For both interventions, participants were allocated 6 capsules that were sealed in an opaque envelope marked with a single letter code by an investigator not involved with data collection. Participants were instructed to consume 1 capsule each morning on an empty stomach for 5 days prior to the experimental trial, and on the morning of this trial (6 days in total). A similar supplementation duration (5 days) with green tea was sufficient in increasing NRF2 activity in rodents (Wang et al. 2015). Each experimental capsule contained 500 mg of green tea extract powder (Taiyo Kagaku, Jiangsu, China). Analysis by the manufacturer confirmed the proportion of total (poly)phenols in the green tea was 96.3% (482 mg), with total catechins 87.4% (421 mg) and EGCG 45.9% (193 mg). Previous research by Hodgson et al. (2014) reported that the catechins in this green tea supplement were bioavailable after an acute bolus. Placebo capsules contained 500 mg of commercially available inulin (Blackburn Distributions, Burnley, UK). Supplements were obtained in powdered form and encapsulated into identical capsules.

Participants attended the laboratory on 3 separate occasions. The first trial was a familiarization session, whereby participants height, body mass, and maximal jump height were collected, and they were familiarized with the eccentric exercise protocol. On the second visit, participants arrived after an overnight fast and resting blood and urine samples were collected. They then consumed a final dose of the supplement, followed 30 min later by a cereal bar (Nature Valley Honey and Oat cereal bar, 42 g, General Mills International, Sárl, Switzerland). 1 h post supplementation, participants performed 100 drop jumps. As in previous studies (Skurvydas et al. 2016; Kamandulis et al. 2017), metabolic stress was minimized by interspersing each jump by a 20 s rest and every 20 jumps with a 2 min rest. Drop jumps involved participants dropping from a 0.6 m box with arms placed on hips to prevent arm swing. Upon landing with two feet on a contact jump mat (JumpMat^™^, FSL Scoreboards, Cookstown, Northern Ireland) they immediately jumped vertically with maximal effort. For each jump, participants were instructed to reach a jump height < 20% of their previously recorded maximal effort jump to ensure near-maximal intensity was achieved. Knee angle in the deceleration phase and technique were visually monitored by a researcher who provided immediate verbal guidance if form declined. Jump height was measured using the contact jump mat, enabling consistent monitoring of exercise intensity. Immediately and 1 h after exercise, blood and urine samples were collected. Participants third visit was 24 h post-exercise, where they returned to the lab fasted for a final blood and urine sample.

### Dietary restrictions and assessment

For 2 days prior and on the second visit, participants recorded their dietary intake with a weighed food diary. For these 3 days, participants were asked to refrain from consuming food and drink high in (poly)phenols, as these foods may activate NRF2 independent of the experimental interventions (Nabavi et al. 2016). A list of restricted foodstuffs was provided to the participant during familiarization. Energy, carbohydrate, fat, protein and Omega-3, vitamin C, D, and E intakes were analyzed using an online dietary analysis software (Nutritics Education v5.81, Nutritics, Dublin, Ireland).

Estimated total (poly)phenol intake (TPI) taken from the food and drink described in the 24 h food diaries were obtained using the Phenol-Explorer v3.6 database (www.phenol-explorer.eu). An advanced search was performed on the database to retrieve mean concentrations of individual (poly)phenols from all food and drink reported. Any sources that contained no or trace amounts of (poly)phenols (i.e., meat) were excluded from analysis. Additionally, some foods recorded on the diaries were not in the database, thus were excluded from analysis. It was reasoned that these foods likely had a low (poly)phenol content since no analysis had been previously conducted. (Poly)phenol intake for a given food or drink source was calculated by multiplying the individual (poly)phenol concentration (determined via chromatography and expressed as mg/100 g food weight) by the quantity of food or drink recorded within the food diary. TPI was calculated as the sum of all (poly)phenol concentrations across the 3 day period. (Poly)phenol content derived from the green tea supplement was not included in this analysis.

### Sample processing and analysis

At each time point, 18 mL of venous blood was collected from the antecubital fossa using a 21-gauge butterfly needle by a trained phlebotomist and drawn into Vacuette containers treated with K^3^ Ethylenediaminetetraacetic acid (K^3^EDTA) (Vacuette, Greiner Bio-One). 4 mL of K^3^EDTA blood was centrifuged at 1500 × *g* for 10 min at 4 °C. Isolated plasma was then pipetted into cryovials and stored at – 80 °C for later analysis. Total and differential leukocyte (neutrophils and monocytes) counts from K^3^EDTA blood was measured using a Yumizen H500 cell counter (Horiba Medical, Montpellier, France) within 5 min of collection. Urine was collected into sterile Falcon tubes (ThermoFisher Scientific, Loughborough, UK), aliquoted into cryovials, then frozen at − 80 °C for later analysis.

### Peripheral blood mononuclear cell isolation and fractionation

10 mL of K^3^EDTA treated blood was diluted at a 1:1 ratio with 1X Dulbecco’s phosphate buffer saline (PBS) (ThermoFisher Scientific, Loughborough, UK) then gently dispensed upon 15 mL Ficoll^®^ paque PLUS (Merck, Darmstadt, Germany) and centrifuged at 400 × *g* for 35 min at 20 °C with the brakes off. PBMCs were harvested and washed twice with PBS at 300 × *g* for 10 min at 20 °C. Following washing, the supernatant was discarded, and the pellet was resuspended in 1 mL RPMI 1640 Complete Medium (Merck, Darmstadt, Germany) before cells were counting by hemocytometry using trypan blue exclusion (0.4%, ThermoFisher Scientific, Massachusetts, United States). PBMCs were aliquoted at 9 × 10^6^ cells/mL and centrifuged at 300 × *g* for 10 min then resuspended in cryoprotectant containing 50% RPMI 1640, 40% FBS, and 10% dimethyl sulfoxide (Merck, Darmstadt, Germany) and frozen at a rate of − 1 °C/min to − 80 °C.

Nuclear proteins were later fractionated from cryopreserved PBMCs using a commercial extraction kit (Nuclear extraction kit, Cat No. 40010, Active Motif, Waterloo, Belgium). Thawed PBMCs were resuspended in 3 mL PBS containing phosphatase inhibitors and centrifuged at 200 × *g* for 10 min at 4 °C. The supernatant was discarded, and the pellet was resuspended in 500 µL hypotonic buffer containing 25 µL detergent then left to swell on ice for 15 min. Once lysed, the cells were centrifuged at 14,000 × *g* for 30 s and the supernatant was removed. The resulting pellet containing the nuclear fraction was resuspended in 50 µL of lysis buffer containing protease inhibitors. It was then vortexed at maximal settings for 10 s and left to incubate on ice for 1 h on an orbital shaker set at 150 rpm. Following incubation, the suspension was vortexed for 30 s at maximal settings then centrifuged at 14,000 × *g* for 10 min. Nuclear fractions were transferred into pre-cooled microcentrifuge tubes and frozen at -80 °C. Protein content of nuclear fractions were measured using a commercial bovine serum albumin assay (Prostain^™^ Protein Quantification Kit, Cat No. 15001, Active Motif, Waterloo, Belgium).

### NRF2/ARE binding

NRF2/ARE binding was measured using a commercially available human NRF2 activity assay (Cat. No. TFEH-NRF2-1, RayBiotech, Georgia, United States) according to manufacturer’s instructions. This method has previously been employed to detect changes in exercise-induced NRF2 activation in rodents (Muthusamy et al. 2012; Ostrom et al. 2021), however this is the first study to employ this method using PBMCs in humans. Nuclear proteins were added to a 96-well plate containing immobilized oligonucleotides possessing the ARE consensus binding site (5′-GTCACAGTACTCAGCAGAATCTG-3′) and left to incubate overnight at 4 °C. Following a wash procedure, primary anti-NRF2 antibodies were added to wells and left to incubate on an orbital shaker set at 150 rpm for 1 h. Each well was washed again, and anti-rabbit horseradish peroxidase conjugated secondary antibodies were added to wells and left to incubate for 1 h. Following colorimetric development, absorbance was read at 450 nm on a Varioskan^™^ LUX multimode microplate reader (ThermoFisher Scientific, Loughborough, UK).

### NRF2 target antioxidant activity

CAT activity was measured in plasma using a commercially available assay (Cat No. 707002, Cayman Chemical, Michigan, USA) according to manufacturer’s instructions. One unit of CAT activity is defined as the number of enzymes causing the formation of 1 nmol of formaldehyde per min (nmol/min/ml). GR activity was measured in plasma using a commercially available assay (Cat No. 703202, Cayman Chemical, Michigan, USA) according to manufacturer’s instructions. One unit of GR activity is defined as the number of enzymes causing the formation of 1 nmol of NAPDH to NADP + per min (nmol/min/ml). The intra-assay CV for CAT and GR was 11.7% and 3.8%, respectively.

### Oxidative DNA damage

Urinary excretion of 8-hydroxy-2′-deoxyguanosine (8-OHdG) was measured using a commercially available competitive monoclonal antibody ELISA (Cat. No. KOA0887, Rockland Immunochemicals, Philadelphia, Pennsylvania, USA) according to manufacturer’s instructions. Urinary creatinine was measured by a commercially available assay (Cat. No. EIACUN, ThermoFisher Scientific, Massachusetts, United States) to account for changes in urine dilution over time. Concentrations were subsequently expressed as ng/mg creatinine. The intra-assay CV for 8-OHdG was 9.8%.

### Statistical analysis

All data are expressed as mean ± SD and were analyzed using IBM SPSS Statistics 27 for Windows (Surrey, UK). Data were checked for normal distribution by inspecting histograms and the Shapiro–Wilk test (*p* > 0.05 for normal distribution). Where data violated normal distribution, log transformations were performed. A 2 (supplement) × 4 (time) mixed model ANOVA was performed on leukocyte counts, DNA damage, antioxidant activity, and NRF2 activity. NRF2/ARE binding was analyzed as fold change from baseline as there were minor differences in protein concentrations between participants. Independent samples t-tests were performed to evaluate group differences in physical characteristics, 3 day energy intake, mean and maximal CMJ height, and TPI. If significant differences for main effects were reported, *post-hoc* tests with Bonferroni corrections were performed to identify the location of differences. Effect sizes for ANOVA analyses were calculated using partial eta squared (ηp^2^: small: 0.01, medium: 0.05, large 0.14 (Cohen 1988)). Where data was missing completely at random for one time point (< 4% of data), expectation–maximization was performed in SPSS to generate parameter estimates (Bennett 2001). If multiple data points were missing for one participant, they were excluded from analysis. If measures did not meet the assumption of sphericity with Mauchly’s test, Greenhouse–Geisser corrections were used. Statistical significance was set at *p* < 0.05 prior to analysis. Graphs were produced using GraphPad Prism (v9.4.1, Boston, USA).

## Results

In total, 24 participants successfully completed the study. No significant differences between groups were reported for physical characteristics or 3 d average energy, macronutrient, and micronutrient intake, and TPI (Table 1). Examination of food diaries revealed all participants adhered to the dietary restrictions and no adverse events were reported in either group.

**Table 1:** Comparison of participants’ physical characteristics and 3-day average dietary intakes

	Green tea	Placebo	*P* value
Physical characteristics			
Age (years)	24 ± 3	23 ± 4	0.63
Height (cm)	178.3 ± 6.7	180.4 ± 5.8	0.43
Weight (kg)	76.7 ± 12.9	80.3 ± 8.1	0.42
Max CMJ height (cm)	38.9 ± 9.1	39.1 ± 6.6	0.97
Mean CMJ height (cm)	36.3 ± 7.9	34.6 ± 6.1	0.57
Dietary energy intake			
Energy (kcal/d)	2378 ± 426	2222 ± 517	0.43
Protein (g/d)	119 ± 25	113 ± 31	0.63
Fat (g/d)	89 ± 22	88 ± 25	0.90
Carbohydrate (g/d)	274 ± 62	242 ± 74	0.26
Omega-3 fatty acid (n-3) (g/d)	1.2 ± 1.2	0.8 ± 0.8	0.32
Vitamin C (mg/d)	73.2 ± 81.7	37.1 ± 22	0.15
Vitamin D (µg/d)	2.5 ± 2.5	3.6 ± 3.8	0.40
Vitamin E (mg/d)	7.9 ± 5.9	6.2 ± 4.3	0.45
TPI (mg/d)	102.7 ± 93.7	67.6 ± 59	0.28

Values are mean ± SD. *n* = 12 per group

*CMJ* counter movement jump

### Exercise intensity

During exercise, all participants recorded a mean CMJ height within 20% of their maximum CMJ height, suggesting intensity was maintained at a near-maximal level. Mean (*p* = 0.58) and maximum (*p* = 0.52) CMJ height was not different between groups (Table 2).

**Table 2:** Comparison of participants’ jump characteristics during exercise

Group	Mean CMJ height (cm)	Max CMJ height (cm)
Green tea	36.3 ± 7.9	38.9 ± 9.1
Placebo	34.6 ± 6.1	39.1 ± 6.6

Values are mean ± SD. *n* = 12 per group

*CMJ* counter movement jump

### Differential leukocyte counts

**Fig. 1:**
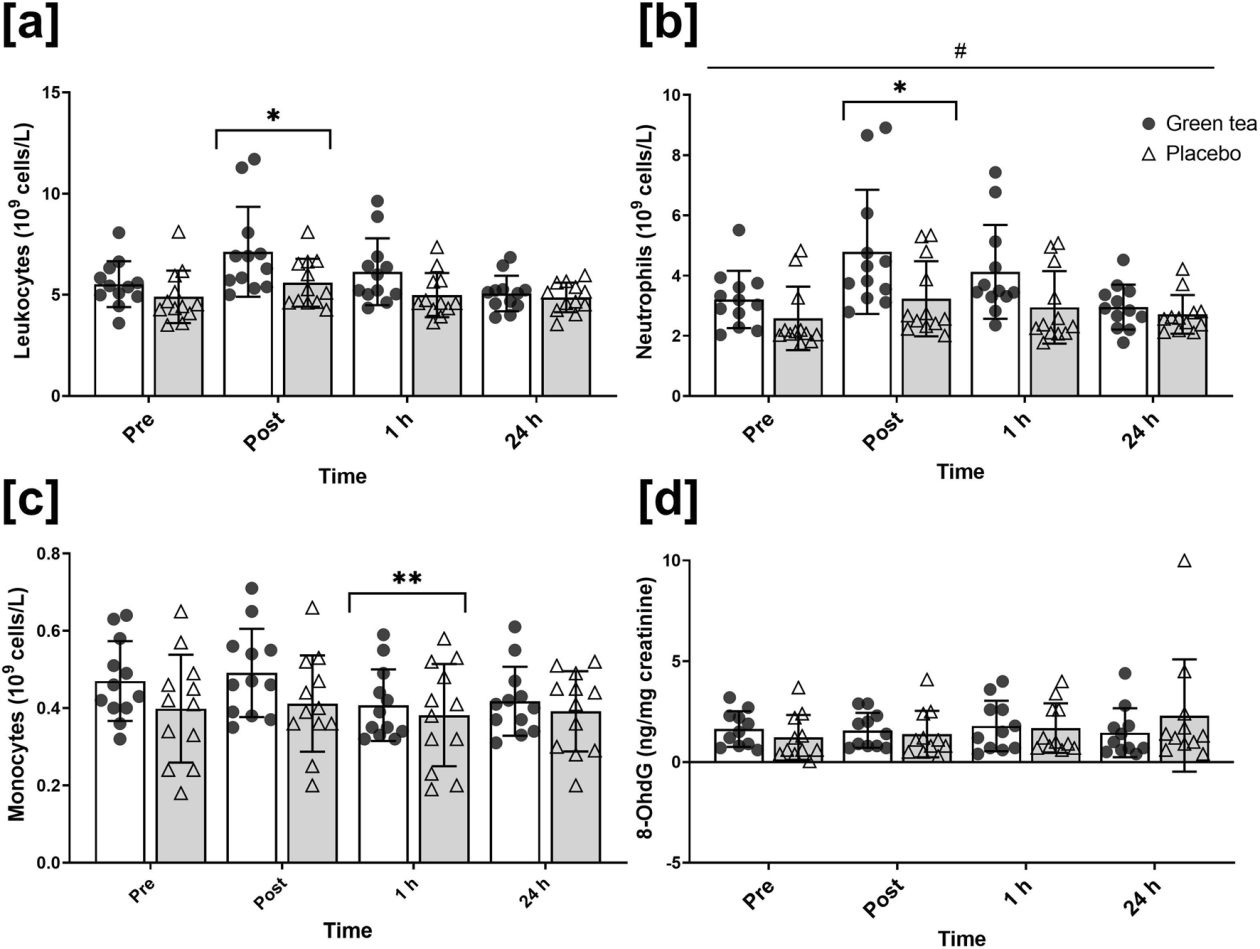
Total leukocyte (**a**), neutrophil (**b**) and monocyte (**c**) concentrations, and 8-OHdG excretion (**d**) measured at pre-, post-, 1 h and 24 h post-exercise. Symbols represent individual values. * significantly different to pre-exercise (*p* < 0.05). ** significantly different to pre- and post-exercise (*p* < 0.05). ^#^ supplement effect (*p* < 0.05). *n* = 12 per group

A main effect for time (*p* < 0.001; ηp^2^ = 0.36) was found for total leukocyte concentration, but no supplement (*p* = 0.06; ηp^2^ = 0.15) or time x supplement (*p* = 0.12; ηp^2^ = 0.09) effect was present (Fig. 1a). A main effect for time (*p* < 0.001; ηp^2^ = 0.39) and supplement (*p* = 0.031; ηp^2^ = 0.20) were reported for neutrophil concentration, but no time x supplement (*p* = 0.056; ηp^2^ = 0.13) effect was found (Fig. 1b). Total leukocytes and neutrophils peaked immediately post-exercise (*p* < 0.001) and returned to resting values by 1 h. Mean neutrophil concentration was significantly higher in the green tea compared to placebo group. A main effect for time was reported for monocyte (*p* < 0.001; ηp^2^ = 0.24) concentration, which were lower at 1 h post-exercise compared to pre-exercise (*p* = 0.02) and post-exercise (monocyte:* p* = 0.002), but no supplement (p = 0.25; ηp^2^ = 0.06) or time x supplement (*p* = 0.11; ηp^2^ = 0.09) effect was found (Fig. 1c).

### DNA oxidation

No main effects for time (*p* = 0.42; ηp^2^ = 0.04), supplement (*p* = 0.64; ηp^2^ = 0.01), or time x supplement (*p* = 0.11, ηp^2^ = 0.1) effect was found for 8-OHdG excretion (Fig. 1d).

### Enzymatic activity of NRF2 targets

**Fig. 2:**
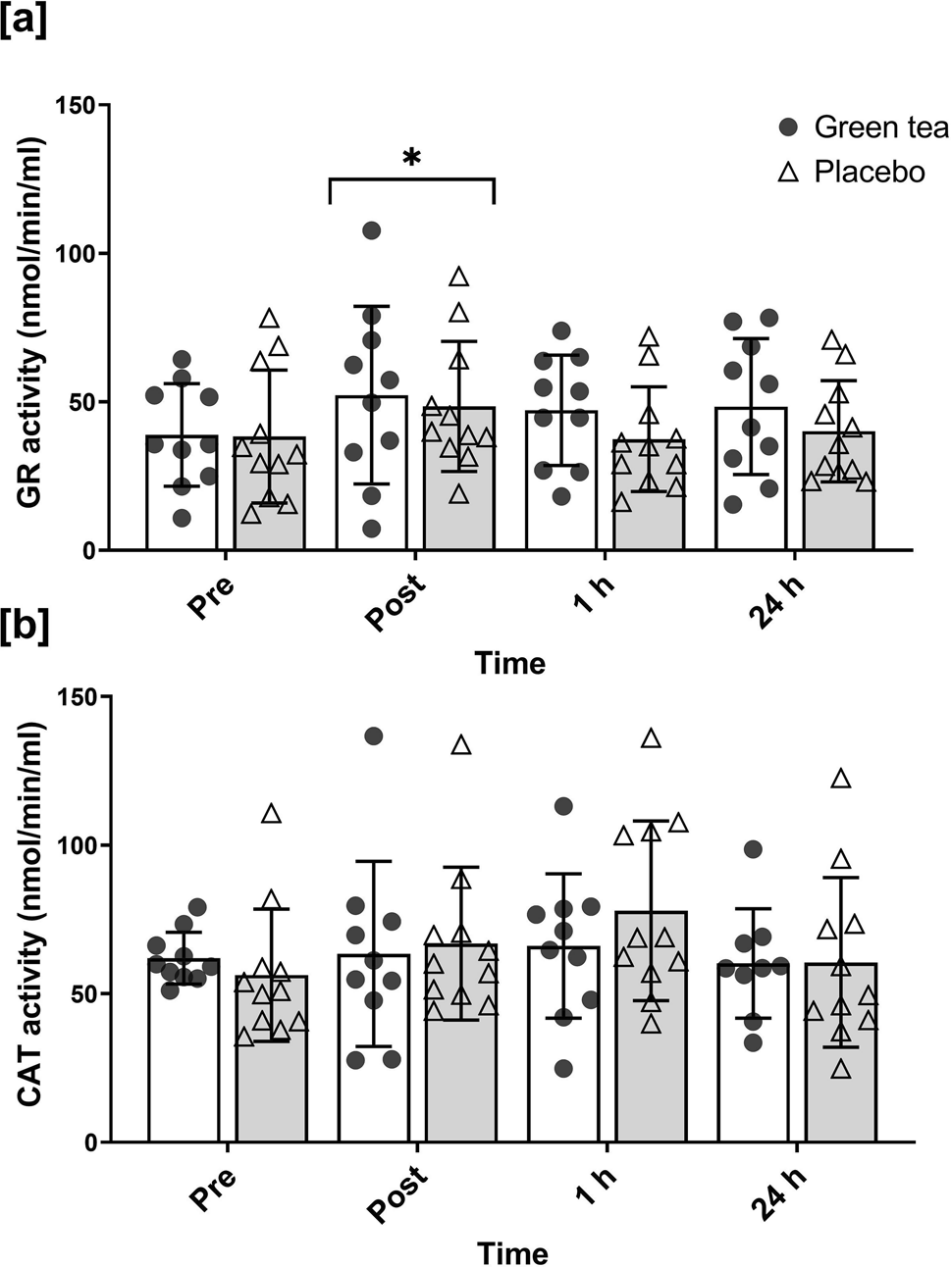
Enzymatic activity of glutathione reductase (GR) (**a**) and catalase (CAT) (**b**) measured at pre-, post- 1 h, and 24 h post-exercise. Symbols represent individual values. *significantly different to pre-exercise (*p* < 0.05). *n* = 10 in green tea group; *n* = 11 in placebo group

Blood plasma could not be collected for *n* = 1 in the green tea group, thus was excluded from this analysis. GR activity increased following exercise (*p* = 0.01; ηp^2^ = 0.17) (Fig. 2a). GR activity increased from pre to post exercise (*p*≤0.01) then returned close to resting values after 1 h. No supplement (*p* = 0.51; ηp^2^ = 0.02) or time x supplement (*p* = 0.50; ηp^2^ = 0.04) effect was found for GR activity. CAT activity did not significantly change, showing no main time (*p* = 0.12; ηp^2^ = 0.10), supplement (*p* = 0.78; ηp^2^≤0.01), or time x supplement (*p* = 0.31; ηp^2^ = 0.06) effect (Fig. 2b).

### NRF2/ARE Binding

**Fig. 3:**
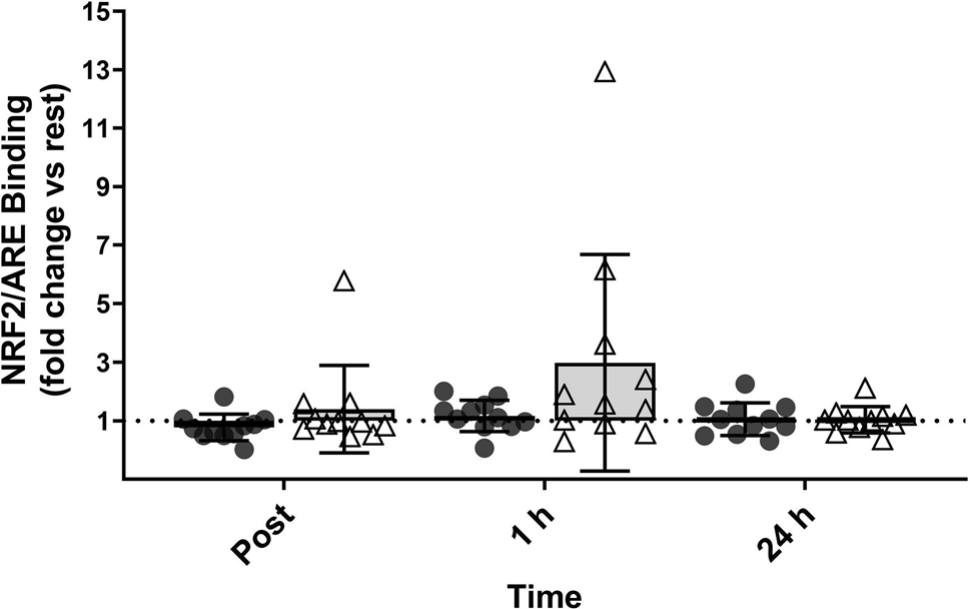
Fold change (vs. rest) of nuclear factor erythroid 2-related factor 2** (**NRF2) binding to antioxidant response element (ARE) oligonucleotides at post-, 1 h, and 24 h post-exercise. Symbols represent individual values. *n* = 11 per group

No main effects for time (*p* = 0.08; ηp^2^ = 0.12), supplement (*p* = 0.18; ηp^2^ = 0.09) or time x supplement (*p* = 0.16; ηp^2^ = 0.09) were found for NRF2/ARE binding (Fig. 3).

## Discussion

This study tested the hypothesis that the combination of eccentric exercise with a 6 days supplementation period of green tea would augment NRF2 activity to a greater extent than eccentric exercise alone, and that this would have favorable downstream effects on NRF2-target antioxidant activity and oxidative damage. Contrary to our hypothesis, we found that neither eccentric exercise, nor the combination of green tea and eccentric exercise, had any significant effect on NRF2 activity.

These findings are consistent with those reported by Wangdi et al. (2021), the only other human study to investigate the synergistic effects of exercise and (poly)phenols on NRF2 activity. In this crossover design study, they reported no changes in skeletal muscle NRF2 protein content immediately or 24 h following repeated eccentric muscle contractions in participants undertaking a 10 day supplementation period with tart cherry juice. Despite this, several studies have reported that when performed without nutritional supplementation, steady-state or high-intensity aerobic exercise increases NRF2 activity (Ballmann et al. 2014; Scott et al. 2015; Done et al. 2016, 2017; Ostrom and Traustadóttir 2020). Thus, the lack of change in our study and Wangdi et al., (2021) could be explained, at least partly, by the different mode of exercise stress employed in comparison to these studies (Done et al. 2016, 2017; Ostrom and Traustadóttir 2020). Indeed, we examined the effects after mechanical stress, as this typically elicits greater skeletal muscle damage than aerobic exercise (Paulsen et al. 2012) and is therefore more often the target of (poly)phenol supplementation (Bowtell and Kelly 2019). Although the eccentric exercise protocol imposed a significant physiological stress, as highlighted by changes to total and differential leukocyte counts comparable to concentric or aerobic exercise, it is possible that the exercise did not sufficiently elevate oxidative stress to the extent required to for canonical activation of NRF2.

Previous research utilizing a similar exercise protocol (100 drop jumps with 20 s rest from 0.5 m box) reported only minor elevations in RONS post-exercise (Kamandulis et al. 2017). We reported no change to 8-OHdG excretion, a biomarker of oxidative DNA damage and a possible reflection of oxidative stress levels post-exercise. Previous research has shown that high intensity exercise, especially aerobic exercise, elevates 8-OHdG excretion (Withee et al. 2017; Tryfidou et al. 2020; Larsen et al. 2020), so perhaps the exercise protocol in our study was not sufficiently intense and/or metabolically demanding to markedly increase DNA oxidation. We also acknowledge that limiting our analysis to 24 h post-exercise means we may have missed changes in 8-OHdG excretion (and possibly our other redox and inflammatory markers) as some studies have shown oxidative damage and inflammation to peak 2–3 days post eccentric exercise (Margaritelis et al. 2015, 2019). In addition, our over-reliance on this single biomarker of oxidative damage is a limitation, since a battery of more sensitive could have been utilized to better detect changes in redox balance (Gomez-Cabrera et al. 2021); nonetheless, these markers are more expensive and technically demanding. Although we anticipated that eccentric exercise may not result in the canonical activation of NRF2, as per Kamandulis et al., (2017) findings, we hypothesized that eccentric exercise would still augment NRF2, but probably non-canonically through the upstream modulation of proteins including ERK1/2. Indeed, activation (phosphorylation) of ERK1/2 occurs following eccentric exercise (Franchi et al. 2014) which can then, in turn, disrupt the NRF2-KEAP1 complex (Verma et al. 2015; Yang et al. 2019). We did not quantify changes in ERK1/2 or related signaling proteins during the study to test this hypothesis and thus acknowledge it as a limitation.

Large inter-individual differences in redox responses at rest and following exercise may also explain the unresponsiveness of NRF2 activity here. Previous research has found that redox related adaptations, such as increased antioxidant activity, are less pronounced in individuals who exhibit low levels of exercise-induced oxidative stress (Margaritelis et al. 2018). This supposed range in redox responses could partly explain the large inter-individual variability we observed in NRF2/ARE binding at 1 h post-exercise. Although we performed a sample size calculation for our analysis, the heterogenous responses would have reduced our statistical power and ultimately limited our ability to detect small effects.

Another discrepancy between our study and previous studies reporting NRF2 activation following exercise is related to the methods used. Across the 5 studies that have measured exercise-induced NRF2 activation in humans, various techniques to quantify changes in NRF2 activity have been implemented. For instance, Ballmann (2014) and Scott (2015) utilized real time PCR to quantify NRF2 gene expression in whole-cell extracts derived from skeletal muscle and leukocytes, respectively. To account for the nuclear localization of NRF2, later work by Done et al. (2016), Done et al., (2017) and Ostrom and Traustadóttir (2020) used western blotting to measure NRF2 protein content in nuclear fractions isolated from PBMCs. To the best of our knowledge, our study employed a novel method to measure the binding of nuclear-bound NRF2 from human PBMCs to ARE oligonucleotides. This technique differs from the other methods as it quantifies the binding of nuclear NRF2 to ARE as opposed to detecting the abundance of NRF2 mRNA expression or protein content. Thus, our method mimics the process involved in NRF2 mediated gene expression, possibly enabling a more precise reflection of NRF2 activity. Previous studies have used the same ARE binding method to detect exercise-induced changes in NRF2 activity, albeit in rodent myocardial and skeletal tissue (Muthusamy et al. 2012; Ostrom et al. 2021). The exercise used in these studies were, however, comparatively more metabolically challenging than ours. For example, Muthusamy et al. (2012) utilized a treadmill run for 2 days (60 min·d^−1^, 14 m/min, 10% incline) whilst Ostrom et al. (2021) implemented a high (100 Hz) electrical muscle stimulation every fourth second for 30 min to imitate exercise stress. As in previous studies, we measured NRF2 activity in PBMCs (Done et al. 2016, 2017; Ostrom and Traustadóttir 2020). PBMCs were preferred to muscle tissue, partly due to their comparative ease of collection, but also because the trauma associated with collecting muscle biopsies can induce immunological responses that could affect NRF2 activity (Malm 2001). Moreover, we reasoned that PBMCs would be more exposed to the electrophilic metabolites of green tea; indeed, there is currently no evidence that (poly)phenol metabolites from green tea reach skeletal tissue, but there is evidence that the specific green tea we used reaches the circulation in small but detectable levels (Hodgson et al. 2014). Muscle samples from single muscle are also limited in that they only represent a small fraction of the musculature and therefore any exercise-induced or nutrition related changes in the non-biopsied muscle fibers are missed (Maeo et al. 2018). Overall, we are satisfied that using this method can be used as an alternative to other time-consuming and expensive methods, such as electrophoretic mobility shift assays and western blotting, to detect changes in NRF2 activity.

Our study did find that the NRF2 gene target, GR, increased by 30% in response to eccentric exercise. Done et al., (2017) similarly reported elevated GR activity following steady-state and high-interval aerobic exercise; however, unlike our study, this was coupled with a concurrent increase in NRF2 expression in PBMCs. Heightened GR activity may suggest that NRF2 activation could have increased in response to eccentric exercise, but that this activation might have occurred elsewhere, such as in the skeletal muscle directly exposed to the mechanical stress. It is unclear why there was a significant elevation in GR whilst CAT remained unchanged. CAT primarily functions by catalyzing elevated H_2_O_2_ levels to water and oxygen (Gebicka and Krych-Madej 2019), so one explanation could be that exercise did not trigger sufficient H_2_O_2_ production to necessitate a rise in CAT. Conversely, it could be that glutathione peroxidase, with reduced glutathione as the substrate, was central to the decomposition of elevated H_2_O_2_ as opposed to CAT. This could also explain the rise in GR activity, since GR is required to recycle oxidized glutathione, which formed from glutathione mediated H_2_O_2_ decomposition, back to reduced glutathione (Couto et al. 2016).

The proposed anti-inflammatory and antioxidant benefits of green tea have been reviewed extensively (Chacko et al. 2010; Chatterjee et al. 2012; Namal Senanayake 2013), with the primary mechanism for these effects largely ascribed to increased NRF2 activation (Christensen and Christensen 2014). However, we found no effect of green tea supplementation on basal or exercise-induced NRF2 activity. Despite green tea and isolated epigallocatechin gallate (EGCG) being reported to increase NRF2 expression in vitro* and *in vivo using rodent models (Chen et al. 2000; Han et al. 2012; Ye et al. 2015; Kanlaya et al. 2016; Tian et al. 2021), these responses had yet to be investigated in humans. The lack of NRF2 activity induced by green tea may be, in part, explained by the selected sample time points. Blood and urinary EGCG concentrations are reported to peak 1–2 h post-ingestion of green tea, tea solids, or purified catechins in humans (Higdon and Frei 2003; Williamson and Manach 2005). Specifically, the green tea extract used in this study was reported to elicit *tmax* of free and conjugated EGCG in plasma 1–2 h post-ingestion of a single bolus, and after 7 days of supplementation (Hodgson et al. 2014). As we only measured NRF2 activity up to 2 h post-ingestion, then not until 24 h later, we may have missed any green tea mediated effects between these time-points. It is also possible that the dose (500 mg·d^−1^) and/or duration of supplementation (6 days) was not sufficient to increase NRF2 activity in humans. Our rationale for the dose and duration was based on several factors. Firstly, we selected a dose of 500 mg·d^−1^ as the daily dose of EGCG (193 mg·d^−1^) was within the maximal daily recommended limit of 338 mg for EGCG (Hu et al. 2018). In addition, a dose of ~ 500 mg·d^−1^ has been repeatedly shown to exert anti-inflammatory and antioxidant effects (Luo et al. 2006; Machado et al. 2018; Sadowska-Krȩpa et al. 2019; Bagheri et al. 2020), as well as reducing markers of exercise-induced muscle damage in humans (da Silva et al. 2018). A 6 day pre-load duration with green tea was also chosen since a similar duration (5 days) of EGCG supplementation (75 mg·kg^−1^) was shown to increase NRF2 expression in rodents (Wang et al. 2015). Continuous intake for several days has previously been proposed to help bypass the relatively short half-life (< 6 h) and low concentration of metabolized EGCG after ingestion (Zhu et al. 2000), thereby leading to an accumulation of EGCG which could elicit transcriptional changes to (poly)phenol-specific membrane transporters and metabolizing enzymes (Scholl et al. 2018). Notwithstanding, future research should investigate whether a higher dose of green tea (poly)phenols, over a longer period, could activate NRF2 in humans.

## Conclusion

Taken together, this study demonstrates that neither mechanically demanding eccentric exercise or a 6 days supplementation period with green tea (poly)phenols increases NRF2 activity in PBMCs. These interventions should therefore be avoided in scenarios where enhancing NRF2 activation is the primary objective. Eccentric exercise may, however, be a useful intervention to induce antioxidative responses, as indicated through elevated GR activity. Further research is warranted to clarify the individual and synergistic response of exercise and (poly)phenol interventions on NRF2 activity.

## Data Availability

Data can be provided at reasonable request from the corresponding author.

## Abbreviations

ARE: Antioxidant response element
ANOVA: Analysis of variance
CAT: Catalase
CMJ: Counter movement jump
CV: Coefficient of variance
Cys: Cysteine
EGCG: Epigallocatechin gallate
ERK: Extracellular signal-regulated kinase
FBS: Fetal bovine serum
GR: Glutathione reductase
KEAP1: Kelch-like ECH-associated protein 1
NRF2: Nuclear factor erythroid 2-related factor 2
PBMCs: Peripheral blood mononuclear cells
PBS: Phosphate buffer sale
RONS: Reactive oxygen and nitrogen species
SD: Standard deviation
TPI: Total (poly)phenol intake
$\dot{V}{\text{O}}_{{{\text{2max}}}}$: Maximal oxygen uptake
8-OHdG: 8-Hydroxy-2′-deoxyguanosine
